# Photo-triggered NO combined with photobiomodulation promotes endothelial repair after balloon angioplasty

**DOI:** 10.1016/j.mtbio.2026.103497

**Published:** 2026-07-29

**Authors:** Shu-jie Jin, Chen-ze Deng, Rou-ye Wang, Fan Jia, Pei-hong Ji, Ya Peng, Ke-feng Ren, Jian-min Liu, You-xiang Wang, Jian Ji

**Affiliations:** a MOE Key Laboratory of Macromolecular Synthesis and Functionalization, Department of Polymer Science and Engineering, Shaoxing Institute, Zhejiang University, Hangzhou, 310058, China; b Key Laboratory of Cardiovascular Intervention and Regenerative Medicine of Zhejiang Province, Department of Cardiology, Sir Run Run Shaw Hospital, Zhejiang University, Hangzhou, 310016, China; c Hangzhou Matrix Medical Technology Co. Ltd, Hangzhou, 311100, China; d Neurosurgery Department of the Third Affiliated Hospital of Soochow University, Changzhou, 213003, China; e Neurovascular Center, Changhai Hospital, Naval Medical University, Shanghai, 200433, China; f State Key Laboratory of Transvascular Implantation Devices, The Second Affiliated Hospital Zhejiang University School of Medicine, Hangzhou, 310009, China

**Keywords:** Photobiomodulation, Photo-triggered drug release, Nitric oxide, Endothelial repair, Balloon angioplasty

## Abstract

Balloon angioplasty, though effective for vascular stenosis, inevitably induces endothelial injury during dilation and clinical strategies to actively promote intimal repair remain lacking. Photobiomodulation (PBM) has demonstrated promising effects in tissue repair through its potent biological regulatory functions. Meanwhile, light is also a particularly appealing tool for drug delivery due to its strong spatiotemporal controllability. In this study, we developed a red light-triggered nitric oxide (NO) releasing coating based on the photosensitive NO donor S-Nitroso-N-acetyl-DL-penicillamine (SNAP). The coating enabled on-demand NO release under red light irradiation, modulating cellular signaling pathways to guide functional endothelial repair. After releasing NO, the SNAP coating significantly promoted endothelial cell (EC) migration and proliferation, while suppressing smooth muscle cell (SMC) activity, resulting in an increase in the EC/SMC ratio by 0.48 in co-culture. In vivo experiments, the combined effect of PBM and light-controlled drug release alleviated vascular inflammation and reduced restenosis (intimal hyperplasia decreased by 63 μm), demonstrating superior therapeutic efficacy compared to PBM alone.

## Graphical abstract

**Figure:**
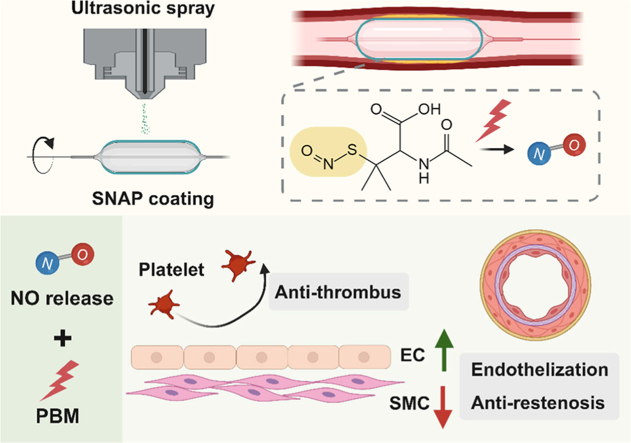

## 1 Introduction

Vascular interventional therapy, owing to its minimal invasiveness and high efficiency, has become a cornerstone in the treatment of cardiovascular diseases [1]. However, balloon dilation during angioplasty inevitably leads to endothelial denudation at the site of injury [2]. This denudation subsequently triggers platelet activation and adhesion, as well as leukocyte infiltration [3], initiating a cascade of inflammatory responses. Cytokines released by activated inflammatory cells further stimulate smooth muscle cells (SMCs) switching to a proliferative phenotype, enhancing SMC proliferation and migration[[4], [5], [6]]. These processes collectively contribute to adverse outcomes including restenosis, thrombus formation and inflammatory reactions [7,8].

Current mainstream clinical strategies to prevent restenosis rely on antiproliferative drugs such as paclitaxel and sirolimus [9,10]. However, potent drugs, while inhibiting smooth muscle cell proliferation, inevitably also suppress endothelial cell repair and delay reendothelialization[[11], [12], [13]]. Despite the clear clinical demand for a therapeutic strategy that selectively enhances endothelial repair while inhibiting smooth muscle cell proliferation, no clinically established approach has yet achieved this desired balance.

Photobiomodulation (PBM), as an emerging non-invasive therapeutic modality, has shown great potential in the field of tissue repair and regeneration [14,15]. Red light/near-infrared light is a commonly used light source in PBM, which can regulate biological behaviors such as cell proliferation, migration, and differentiation[[16], [17], [18]]. Previous studies have shown a decreased restenosis rate in coronary heart disease patients who received red laser PBM treatment [19,20]. The primary mechanism involves absorption of photons by cytochrome *c* oxidase (Cox), a key mitochondrial enzyme. This photon absorption dissociates inhibitory nitric oxide (NO) from Cox, thereby restoring mitochondrial respiration, increasing electron transport and elevating ATP production [21]. Then they activate downstream signaling pathways and transcription factors, leading to cell activity changes. Nevertheless, the therapeutic efficacy of PBM alone can be modest. Combining PBM with on-demand drug release technologies may offer a synergistic approach to enhance efficacy while minimizing systemic side effects.

During balloon angioplasty, balloon inflation typically lasts only dozens of seconds, imposing a narrow therapeutic window for drug delivery. Recent advances have focused on developing smart coatings that respond to the local injury microenvironment (such as ROS, pH, specific enzymes)[[22], [23], [24], [25]], or that enable drug release triggered by external stimuli such as light or ultrasound [[26], [27], [28]]. Among these, light is a particularly appealing tool for on-demand drug delivery due to its strong spatiotemporal controllability, enabling precise drug release through photochemical, photothermal, and photoisomerization-mediated reactions.

Compared with cytotoxic antiproliferative drugs, biologic molecular drugs have relatively safer metabolism and lower overall toxicity. Various bioactive substances such as gaseous signaling molecules[[29], [30], [31]], antibodies [32], and cells [33,34] have been explored for cardiovascular therapy. Nitric oxide (NO), a naturally occurring signaling molecule, plays a pivotal role in vascular homeostasis. NO promotes functional endothelial repair by regulating cellular signaling pathways, while also inhibiting platelet aggregation and thrombus formation, exerting anti-inflammatory effects, and suppressing excessive SMC proliferation and migration [35,36]. S-Nitrosothiols (RSNO) are widely employed as NO donors due to their favorable biocompatibility [37]. Representative RSNOs, including S-nitrosoglutathione (GSNO) and S-nitroso-N-acetyl-DL-penicillamine (SNAP), are susceptible to decomposition when triggered by heat, light, or metal ions [38,39]. It can be directly incorporated into the polymer matrix [40], or fixed on the surface of the material through physical adsorption/chemical grafting to exert the properties of anti-thrombosis and promote endothelial repair.

While NO-releasing coatings for vascular stents have been extensively reviewed [41], the application of such coatings specifically on balloon catheters for acute vascular injury during angioplasty, particularly in combination with photobiomodulation, remains underexplored. Herein, we developed a red light-triggered NO releasing coating based on the photosensitive NO donor S-Nitroso-N-acetyl-DL-penicillamine (SNAP) [42,43] and excipient shellac. Shellac, a naturally derived, FDA-approved polymer with established biocompatibility and clinical use in drug-eluting balloons [44], provides a hydrophobic microenvironment that stabilizes SNAP by preventing premature dissolution. This coating is designed specifically for balloon catheter surfaces. Our strategy integrates two complementary mechanisms: (i) photobiomodulation, and (ii) photo-triggered NO release from the coating, which accelerates vascular endothelial repair and suppresses restenosis. Our PBM-compatible NO release balloon catheter coating significantly enhanced endothelial repair both in vitro and in vivo, demonstrating a synergistic approach for post-angioplasty vascular healing.

## 2 Materials and methods

### 2.1 Materials

S-Nitroso-N-acetyl-DL-penicillamine (SNAP, Aladdin, Shanghai, China, S131283, purity ≥97% by HPLC) was stored at −20°C in the dark prior to use. Shellac was purchased from Shanghai Haohong Biomedical Technology (Shanghai, China, 1268826, CP grade, CAS: 9000-59-3). Endothelial cell medium (ECM), smooth muscle cell medium (SMCM), and fetal bovine serum (FBS) were purchased from ScienCell Research Laboratories (Carlsbad, CA, USA). CellTracker Green CMFDA (C2925) and CellTracker Red CMTPX (A66435) were purchased from Thermo Fisher Scientific (Waltham, MA, USA). Anhydrous ethanol was obtained from Sinopharm Chemical Reagent (Shanghai, China). Ultrapure water was produced by a Millipore Milli-Q water purification system (Aquaplore). PBM light source was a 630-640 nm LED array purchased from Aijia electronic technology (Xuzhou, China).

### 2.2 Preparation of the SNAP coating

Substrate preparation: PDMS prepolymer and curing agent are evenly mixed at a weight ratio of 10:1 (prepolymer to curing agent), cured in an oven at 80°C for 2 h. The TPU substrate and PDMS substrate were ultrasonically washed in ethanol for 5 min, and then were blown dry by nitrogen for later use.

Ultrasonic spraying to prepare the coating: SNAP and excipient shellac were dissolved in ethanol at a mass ratio of 3:1 to prepare ultrasonic spraying solution. The solution was uniformly sprayed on the experimental substrate and the surface of the balloon catheter (diameter 1.75 mm, length 20 mm) by ultrasonic spraying technology (DP30, Siansonic, China) to construct SNAP coatings with different drug loading densities (1, 3, 5, 10, 20 μg/mm^2^). All processes were carried out in a dark environment.

### 2.3 Structural and NO release characterization of coatings

Attenuated total reflection Fourier transform infrared spectroscopy (FTIR-ATR, Nicolet 6700, USA) and X-ray photoelectron spectroscopy (XPS, K-alpha, USA) were used to characterize the chemical structure of the coating. Scanning electron microscopy (SEM, Hitachi SU8600, Japan) was used to characterize the topography and thickness of the coating.

NO release detection: The SNAP/shellac coating prepared after spraying was placed in 1 mL water and 3.6 J/cm^2^ (20 mW/cm^2^, 3 min) red light irradiation was applied. The NO release quantity in different drug loading density SNAP coatings was detected by NO detection kit (S0021S, Beyotime, China). NO concentration released was normalized to a sample surface area.

### 2.4 Characterization of anticoagulant properties of coatings

Platelet adhesion experiment: Fresh rabbit blood was taken from New Zealand rabbits, and the anticoagulant sodium citrate was added at a ratio of 9:1 (v/v). Rabbit blood was centrifuged in a centrifuge at 1500 rpm for 15 min to obtain platelet-containing plasma. 100 μL drops were added to the surface of 8 mm diameter circular samples. The samples were incubated at 37°C for 2 h and then washed with PBS 3 times, fixed with 2.5% glutaraldehyde solution for 30 min and then washed 3 times. The samples were then dehydrated step by step with aqueous ethanol solutions (20%, 40%, 50%, 60%, 70%, 80%, 90%, 100%). The adhesion of platelets on the surface of the sample was observed by SEM, and the number of platelets was counted by ImageJ.

Whole blood coagulation experiment: Add 10% 0.2 M CaCl_2_ solution to blood containing sodium citrate anticoagulant. 100 μL blood was added to the surface of a circular sample with a diameter of 8 mm and incubated at 37°C for 5 min, 10 min, and 15 min. The samples were then gently washed in PBS and the clotting on the sample surface was recorded.

### 2.5 Cell culture

Human umbilical vein endothelial cells (HUVEC) and the human arterial smooth muscle cells (HASMC) at passages 3 to 7 were used in our experiments and were cultured at 37°C in a humidified atmosphere containing 5% CO_2_. The culture medium was changed every 2 or 3 days to maintain optimal growth conditions.

### 2.6 Proliferation, migration, and co-culture of ECs and SMCs

Cell proliferation: Cells were seeded at a density of 5000 cells/cm^2^ in 48-well cell culture plates and covered with different drug loading densities SNAP coatings (5 mm✕5 mm, 500 μL medium). Then, red light irradiation was performed at 20 mW/cm^2^ for 3 min. After the irradiation, the coating sample was removed, and the medium was changed to continue the culture. The cells were stained with CellTracker. Cell numbers were then counted at 24 h and 48 h after coating treatment to observe the effect of the SNAP coating on cell proliferation. In addition, CCK8 was used to detect the cell activity after coating treatment.

Cell migration: Cells were seeded at a density of 100,000 cells/well in 12-well cell culture plate. Once the cells were basically covered, a 10 μL pipette tip was used to create a vertical scratch in the middle of each well. The SNAP coating (1 cm✕1 cm, 2 mL medium) was applied to the cell surface and red light was applied. The cells were washed with PBS to remove dead cells, ensuring a clear gap line was visible. Low serum (2%) DMEM medium (Gibco, USA) was used to minimize the influence of cell proliferation. The gap width was observed under a microscope (DS-Ri2, Nikon, Japan) at 0 h, 6 h, and 24 h post-irradiation to calculate the cell migration ratio.

Co-culture: ECs and SMCs were stained with CellTracker Green CMFDA and CellTracker Red CMTPX respectively and then seeded in 48-well plates at a density of 5000 cells/cm^2^ (10,000 cells/cm^2^ total). Cells were covered with SNAP coating on the surface and received red light irradiation. After co-cultured for 24 h, fluorescence images were taken to count cell densities.

### 2.7 Angiogenesis and endothelial function testing

Angiogenesis: The matrix gel (Biosharp, China) was placed on ice and melted at 4°C, then diluted with ECM at a ratio of 2:1 (matrix gel: ECM). A 50 μL aliquot of the mixed solution was added per well and incubated at 37°C for 30 min. The stained endothelial cells were seeded into the plate at a density of 1 × 10^4^ cells/well, followed by treatment with the SNAP coating and red light irradiation. The vascular network formation of endothelial cells was observed after 4 h.

ELISA detection of cGMP and PGI2: The levels of cyclic guanosine monophosphate (cGMP) and prostacyclin (PGI2) were measured using ELISA kits. Endothelial cells were treated with SNAP coating and PBM, and the expression and secretion of cGMP and PGI2 in endothelial cells were detected by cGMP ELISA Kit (E-EL-0083, Elabscience, China) and PGI2 ELISA Kit (E-EL-0022, Elabscience, China) after 24 h.

### 2.8 RAW 264.7 cell polarization detection

After seeding RAW 264.7 macrophages, macrophage M1 polarization was induced with 1 μg/ml lipopolysaccharide (LPS) solution. The cells were then treated with the SNAP coating and exposed to red light irradiation. After 24 h, the cells were digested and centrifuged. The pellet was resuspended in 1 mL of FACS solution (PBS + 2% FBS + 2 mM EDTA) and centrifuged at 1000 rpm for 5 min. The supernatant was discarded, and the cells were resuspended in 100 μL FACS solution. 1 μL Fc blocking antibody (C1755S, Beyotime, China) was added to the suspended cells and incubation was carried out on ice for 20 min. After blocking, 400 μL FACS solution was added. Then centrifuged at 1000 rpm for 5 min and the supernatant was discarded. CD86 (99879S, Cell Signaling Technology, USA) and CD206 (141,706, BioLegend, USA) fluorescent primary antibody staining solutions were diluted at a ratio of 1:200 in FACS buffer. Incubation was performed on ice away from light for 30 min. After washing three times with PBS buffer, the cells were resuspended in 200 μL PBS. Cell fluorescence and polarization status were analyzed by a flow cytometer (CytoFLEX, Beckman Coulter, USA).

### 2.9 Rat abdominal aorta model

Sprague–Dawley (SD) rats were obtained from the Zhejiang Center of Laboratory Animals (ZJCLA, Hangzhou, China). All animal experiments were approved by the Institutional Animal Care and Use Committee (IACUC) of ZJCLA. The approval number for these experiments was ZJCLA-IACUC-20011415.

Balloon-mediated vascular injury was induced in the SD rat abdominal aorta. The rats were anesthetized by isoflurane inhalation (5% for induction, then 1.5% for maintenance). A longitudinal incision was made and the abdominal aorta was dissected. The 1.75 mm diameter balloon (MATRIXMEDICAL, China) was inserted into the abdominal aorta, inflated with 4 atm pressure, and kept at this pressure for 3 min. For the PBM-treated and SNAP coating group, a red light fiber within the balloon was activated to provide 20 mW/cm^2^ irradiation for 3 min. Rats were randomly assigned to different groups, with 4 rats in each group. After the deflation and withdrawal of the balloon, the surgical site was closed with sutures. No anticoagulation or antiplatelet treatments were given before and after the surgeries. At 3 days and 28 days post-surgery, the rats were anesthetized, and the abdominal aortas were harvested for analysis.

### 2.10 Histological staining

Vascular samples were fixed with 4% paraformaldehyde at room temperature overnight and the fixed samples were embedded in paraffin and sectioned. Slides were then stained with HE, Masson, EVG to observe its morphology.

### 2.11 Tissue immunofluorescence staining

Tissue sections were first blocked with 3% bovine serum albumin (BSA, Sigma-Aldrich, USA) solution for 30 min and then incubated with the following primary antibodies in BSA solution overnight at 4°C: CD68 (HKA50068, HaoKe, China), CD86 (13395-1-AP, Proteintech, China), CD206 (18704-1-AP, Proteintech, China), CD31 (ab182981, Abcam, UK), Cyclin D1 (BX50071, Biolynx, China), Ki67 (B11002R, HaoKe, China) and α-SMA (HKA50059, HaoKe, China). After that, samples were washed with PBS and incubated with goat anti-rabbit secondary antibody or goat anti-mouse antibody for 1 h at room temperature. Nuclei were counterstained with DAPI. The stained samples were scanned with a fluorescence microscope (Nikon).

### 2.12 Western Blot and qPCR test

Cells were harvested and centrifuged 24 h post PBM and SNAP coating treatment. Tissue samples are rapidly frozen in liquid nitrogen to be used for subsequent experiments. The samples were transported by dry ice. Western Blot and qPCR analyses were outsourced to Newbe Biology (Hangzhou, China). Antibodies used in WB: sGC (ab172480, Abcam, UK), PKG (3248, Cell Signaling Technology, USA), p-VASP (3114, Cell Signaling Technology, USA), p21 (ab109199, Abcam, UK), Cyclin D1 (ab16663, Abcam, UK), p-Rb (8516, Cell Signaling Technology, USA), p-eNOS (PA5-104858, Invitrogen, USA), vWF (11778-1-AP, Proteintech, China), VE-cadherin (36-1900, Invitrogen, USA), β-actin (ab8266, Abcam, UK).

### 2.13 Transcriptomics analysis

Total RNA was isolated from frozen tissues using TRIzol reagent. mRNA-sequencing and data analysis was subjected to Majorbio (Shanghai, China). Differentially expressed genes (DEGs) were identified using DESeq2. Genes with p < 0.05 and |fold change| ≥ 2 were considered significant.

### 2.14 Statistical analysis

All data were obtained from at least three parallel samples per condition in each experiment and are expressed as mean ± standard deviation (SD). Statistical significance between different groups was assessed using one-way ANOVA followed by Tukey's post hoc test. The significance level was set at *p < 0.05, **p < 0.01 and ***p < 0.001.

## 3 Results

### 3.1 Construction and Characterization of Coatings

**Fig. 1:**
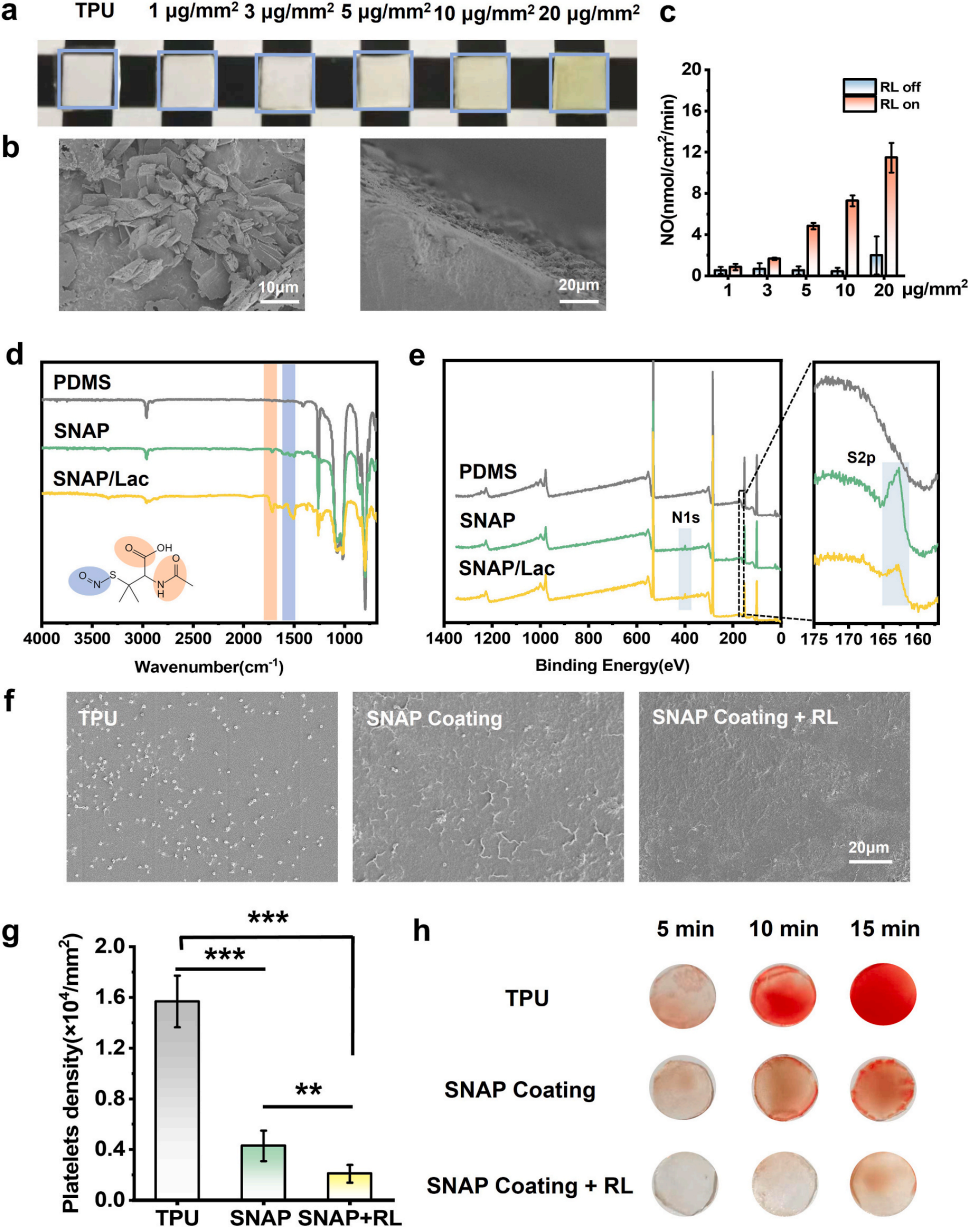
**Construction and Characterization of Coatings**. (a) Images of SNAP/Shellac coatings with different drug-loading densities. (b) SEM images of the coating surface and cross-section. (c) NO release capacity of different drug-loading densities coatings under red light irradiation (n = 3). (d) Infrared spectroscopic analysis of the substrate and coating surface. (e) XPS analysis of the substrate and coating surface. (f) SEM images of platelets adhesion on TPU, SNAP coating, and light-irradiated SNAP coating surfaces. (g) Quantitative analysis of platelets adhesion (n = 9), ∗p < 0.05, ∗∗p < 0.01, ∗∗∗p < 0.001. (h) Surface images after whole blood incubation at different time points. (For interpretation of the references to color in this figure legend, the reader is referred to the Web version of this article.)

As a classic and widely studied NO donor of nitrosothiols (RSNOs), SNAP is a commonly used donor for NO releasing coatings in biomaterials. As shown in Fig. 1a, we prepared a SNAP/shellac coating on the substrate surface by ultrasonic spraying, and the color of the coating became more and more pronounced as the drug-loaded mass density increased. Through scanning electron microscopy, we observed the crystal morphology of SNAP particles, presenting needle-like and flake-like forms (Fig. 1b). Under red light irradiation at 20 mW/cm^2^ for 3 min, the amount of NO released increased with the increase of SNAP content on the surface. The average NO release was 4.8 ± 0.3 nmol/cm^2^/min for a 5 μg/mm^2^ SNAP coating, and up to 11.5 ± 1.4 nmol/cm^2^/min for a 20 μg/mm^2^ coating after irradiation (Fig. 1c). There was no obvious NO release without light. The coating thickness varied with the SNAP loading density; at a loading density of 5 μg/mm^2^, the thickness was approximately 10 μm (Supplementary Fig. 1). 5 μg/mm^2^ SNAP/lac coating leaching into the PBS buffer was quantified by UV-Vis spectrophotometry at 340 nm (Supplementary Fig. 2). We found that SNAP did indeed have some leaching, showing stability still needed to be improved. The coating exhibited a water contact angle of approximately 64°, placing it in the intermediate wettability range (Supplementary Fig. 3).

Due to the large number of infrared characteristic peaks of the TPU substrate, there will be interference when comparing the infrared spectra of the coating, so we chose the PDMS substrate. As shown in Fig. 1d, the PDMS substrate had no characteristic peak at 1800-1600 cm^−1^, while the 5 μg/mm^2^ pure SNAP coating and SNAP/shellac coating had characteristic peaks at 1720 cm^−1^, corresponding to the stretching vibration of the carbonyl group. There were also characteristic peaks around 1550 cm^−1^, which came from the -SNO bond. In the XPS spectra (Fig. 1e), the characteristic peak of N element was shown at 400 eV which was the 1s orbital transition of N, and the characteristic peak of S element was shown at 163 eV which was the 2p orbital transition of S. Experimental results from both infrared spectra and XPS spectra showed successful coating preparation.

The primary challenge of implantable blood-contacting materials is thrombosis formation. As one of the powerful endogenous anticoagulant molecules in the human body, NO can maintain the normal flow of blood and play an anticoagulant role through the comprehensive pathways. On the TPU substrate without SNAP coating, the adhesion density of platelets after 2 h incubation reached to 1.57 ± 0.20 × 10^4^/mm^2^. The adhesion density of platelets on the SNAP coating without light was 0.43 ± 0.12 × 10^4^/mm^2^ while platelet adhesion density decreased to 0.21 ± 0.07 × 10^4^/mm^2^ after NO release triggered by red light, nearly 8 times lower than the substrate (Fig. 1f and g). The coating exhibited potent antiplatelet adhesion ability, supporting the antithrombotic potential for post-angioplasty applications [45,46]. In the whole blood coagulation experiment, no coagulation occurred at 5 min. After 10 min, the TPU substrate and the SNAP coating without red light irradiation had begun to coagulate. After 15 min, the TPU substrate coagulated significantly, the SNAP coating without irradiation had partial coagulation, but no coagulation occurred after the release of NO (Fig. 1h). Both platelet adhesion and whole blood coagulation reflected the excellent anticoagulant ability of SNAP coating after NO release triggered by light.

### 3.2 The influence of SNAP coating on endothelial cells

**Fig. 2:**
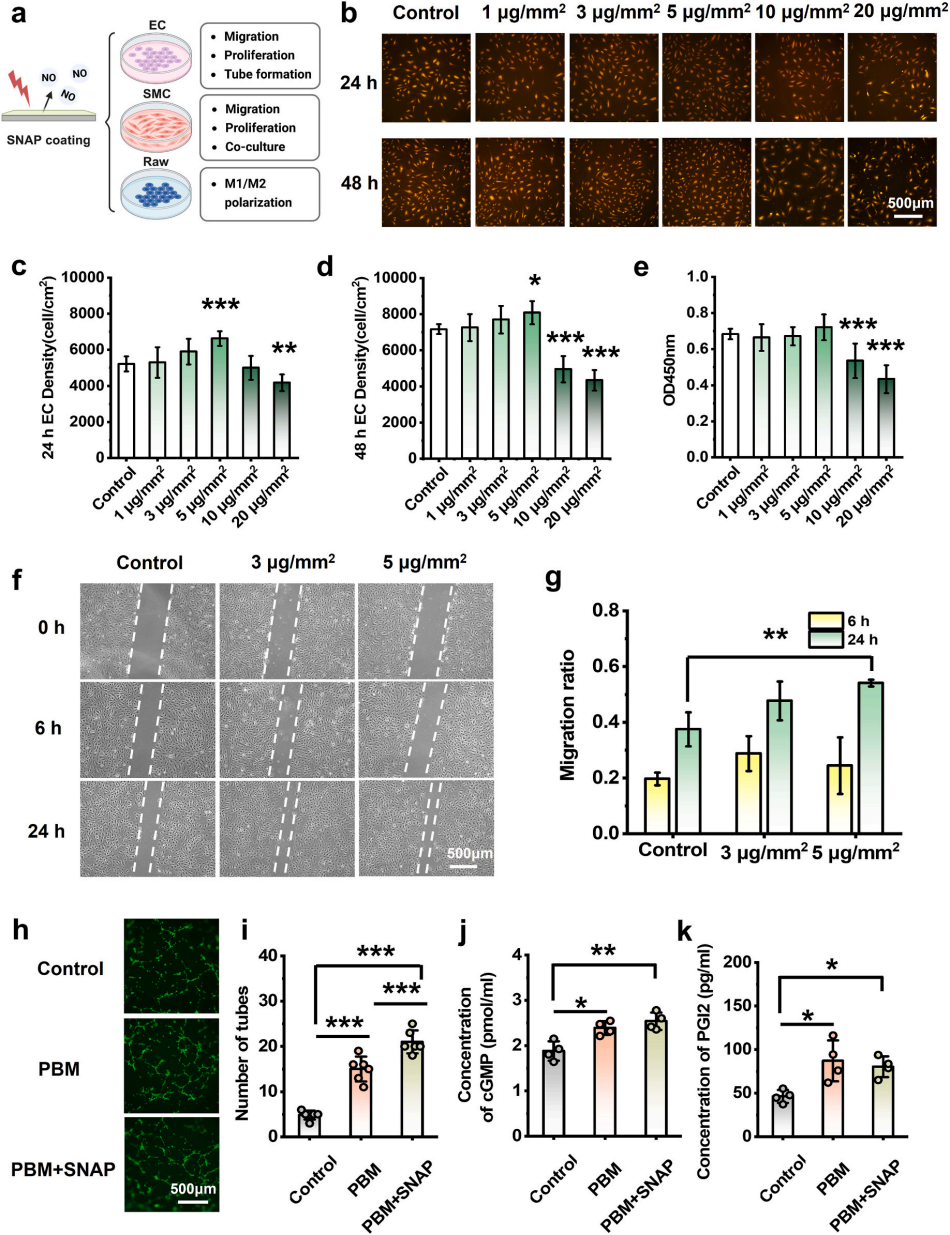
**The influence of SNAP coating on ECs**. (a) Schematic diagram of the effect of SNAP coating on cells. (b) Proliferation images of ECs after NO release from different drug loading densities SNAP coatings with light irradiation. (c) EC density after 24 h (n = 9). (d) EC density after 48 h (n = 9). (e) EC activity after 48 h (n = 9). (f) EC migration under no coating, 3 μg/mm^2^, 5 μg/mm^2^ SNAP coating with red light irradiation. (g) EC migration ratio after 6 h and 24 h (n = 4). (h) Images of tube formation after PBM and coating treatment. (i) Number of tube formations (n = 6). (j) Expression of cGMP in EC (n = 4). (k) Secretion of PGI2 in EC (n = 4). *p < 0.05, **p < 0.01, ***p < 0.001. (For interpretation of the references to color in this figure legend, the reader is referred to the Web version of this article.)

To investigate the effect of SNAP coating on endothelial repair, we prepared SNAP coatings with different drug-loading densities covering the cell surface and applied red light irradiation to promote NO release (Fig. 2a). We conducted endothelial cell proliferation and migration assays to identify an appropriate SNAP drug dosage suitable for vascular repair.

In the EC proliferation experiment, SNAP coatings with low drug-loading densities, such as 1 μg/mm^2^ and 3 μg/mm^2^, did not exert a particularly significant effect on endothelial proliferation. At 5 μg/mm^2^, the SNAP coating promoted endothelial proliferation: after 24 h, the endothelial cell density increased from 5.22 ± 0.42 × 10^3^ cells/cm^2^ (uncoated control) to 6.62 ± 0.41 × 10^3^ cells/cm^2^; after 48 h, it increased from 7.17 ± 0.28 × 10^3^ cells/cm^2^ to 8.08 ± 0.64 × 10^3^ cells/cm^2^. In contrast, when the SNAP content was further increased to 10 μg/mm^2^ and 20 μg/mm^2^, it induced endothelial apoptosis and exacerbated endothelial injury, which may be due to excessively high NO concentrations released (Fig. 2b–d). CCK8 results showed that after treatment with the 10 μg/mm^2^ coating, the absorbance at 450 nm decreased from 0.68 ± 0.03 to 0.54 ± 0.10; at 20 μg/mm^2^, the inhibitory effect on activity was more pronounced, with absorbance dropping to 0.43 ± 0.08, corresponding to a 37% inhibition of cell viability (Fig. 2e). As a free radical and oxidant, high-concentration NO was prone to cause direct cellular damage. The biological effects of NO followed a biphasic dose-response pattern [47,48], with an appropriate therapeutic window existing. Through the EC proliferation experiment, we found that a 5 μg/mm^2^ SNAP coating appeared relatively suitable, as it could exert a certain pro-endothelial proliferative effect without inducing cytotoxicity.

As shown in Fig. 2f, NO release also promotes the migratory capacity of endothelial cells. After the addition of SNAP coating, the scratch width of ECs was narrower with greater coverage. After 24 h, the migration ratio of endothelial cells increased from 37% ± 6% (control group without coating) to 48% ± 7% (3 μg/mm^2^ SNAP coating) and 54% ± 1% (5 μg/mm^2^ SNAP coating) (Fig. 2g). Whether endothelial cells can rapidly cover the exposed wound surface after vascular injury is crucial for achieving re-endothelialization. As a biofunctional molecule that promotes endothelial cell repair, NO has increasing applications in surface modification strategies of medical devices such as scaffolds and balloons[[49], [50], [51]].

Tube formation is a core in vitro assay for evaluating endothelial cell (EC) function and studying angiogenesis and repair. It mimics the key step of ECs assembling into three-dimensional tubular networks. As shown in Fig. 2h, after red light triggered NO release, the tube-forming ability of ECs was enhanced with the number of tubes increasing from 5 ± 1 to 21 ± 3, a greater improvement compared to PBM alone (tube number: 15 ± 3) (Fig. 2i). The enhanced tube-forming capacity was beneficial for re-endothelialization and angiogenesis.

NO can activate soluble guanylate cyclase (sGC), thereby increasing intracellular cyclic guanosine monophosphate (cGMP) levels. cGMP, as a central second messenger in EC function, maintains vascular dilation and barrier integrity [35]. Following light-induced NO release, cGMP expression also increased, with concentration rising from 1.88 ± 0.21 pmol/mL to 2.54 ± 0.19 pmol/mL (2.39 ± 0.15 pmol/mL with PBM alone) (Fig. 2j). In addition to cGMP, prostacyclin (PGI2) also exhibited vasodilatory and antiplatelet aggregatory effects. After treating ECs with the coating, the secreted PGI2 concentrations were enhanced to 80.28 ± 11.94 pg/mL from 46.21 ± 7.44 pg/mL (Fig. 2k). The increased expression of cGMP and PGI2 also demonstrated enhanced EC functions such as antiplatelet activity and vasodilation, which maintained vascular homeostasis.

### 3.3 The influence of SNAP coating on smooth muscle cells and RAW 264.7

**Fig. 3:**
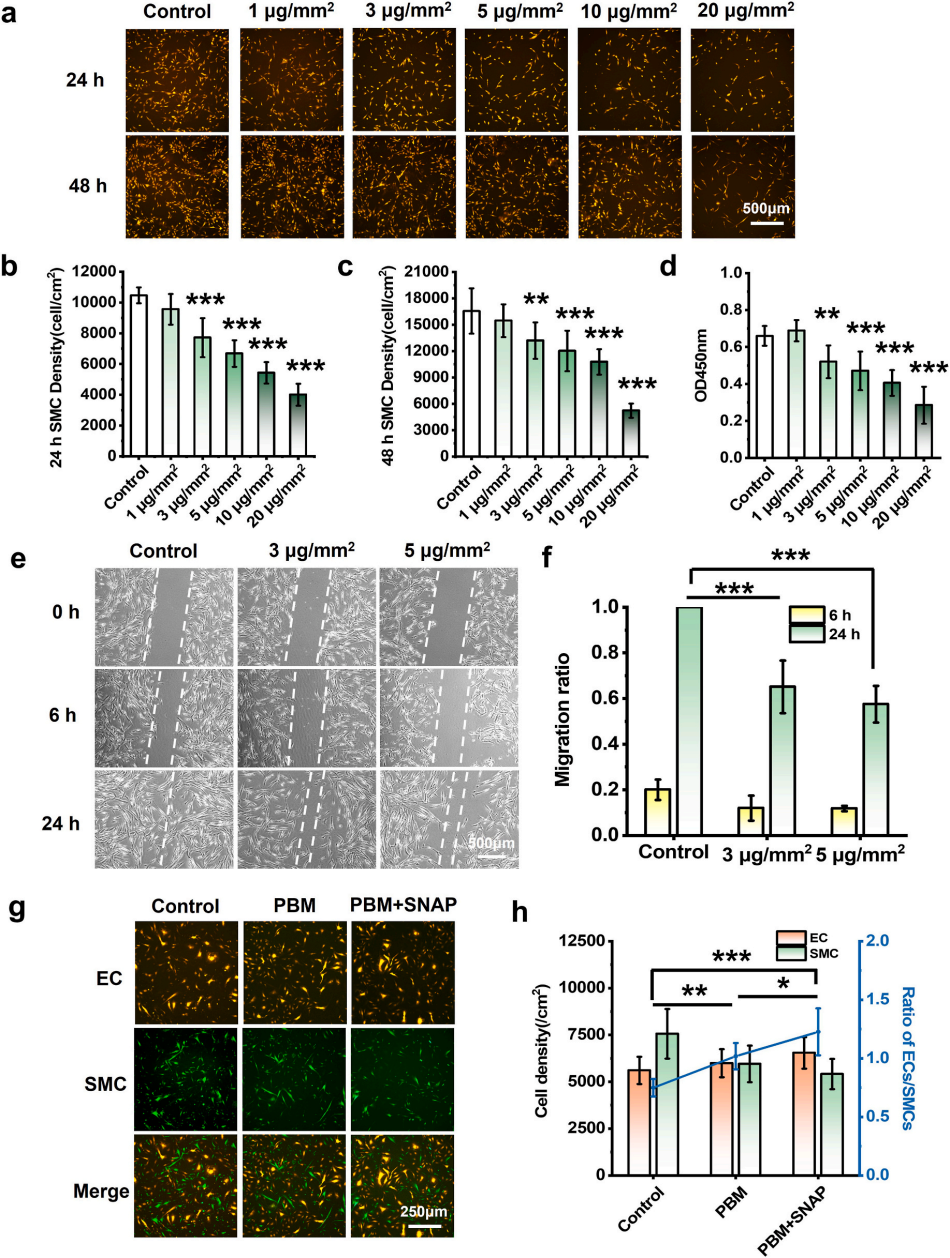
**The influence of SNAP coating on SMCs and co-culture.** (a) Proliferation images of SMCs after NO release from different drug loading densities SNAP coatings with light irradiation. (b) SMC density after 24 h (n = 9). (c) SMC density after 48 h (n = 9). (d) SMC activity after 48 h (n = 9). (e) SMC migration under no coating, 3 μg/mm^2^, 5 μg/mm^2^ SNAP coating with red light irradiation. (f) SMC migration ratio after 6 h and 24 h (n = 4). (g) Co-culture of ECs and SMCs after PBM and coating treatment (orange: ECs, green: SMCs). (h) Quantification of the cell density and the ratio of ECs/SMCs (n = 9), ∗p < 0.05, ∗∗p < 0.01, ∗∗∗p < 0.001. (For interpretation of the references to color in this figure legend, the reader is referred to the Web version of this article.)

NO also plays a key role in regulating the proliferation of smooth muscle cells. We found that red light irradiating SNAP coating significantly inhibited the growth of smooth muscle cells and promoted their apoptosis (Fig. 3a). The inhibitory effect increased as the SNAP content increased. Without the coating, the number of smooth muscle cells proliferated to 1.05 ± 0.05 × 10^4^ cells/cm^2^ after 24 h. With SNAP coatings of 1, 3, 5, 10, and 20 μg/mm^2^, the cell counts decreased to 9.55 ± 1.00, 7.71 ± 1.27, 6.68 ± 0.87, 5.42 ± 0.70, and 4.00 ± 0.71 × 10^3^ cells/cm^2^, respectively (Fig. 3b). After 48 h, the number and activity of SMCs also showed a similar downward trend (Fig. 3c and d). If red light was not applied, there was no significant effect on the cell activity of ECs and SMCs without NO release (Supplementary Fig. 4). NO had a significant effect on slowing the proliferation rate of SMCs and inducing their apoptosis, which was positive for reducing restenosis.

As shown in Fig. 3e, NO release also inhibited the migratory capacity of SMCs. After the addition of SNAP coating, the migratory capacity of SMCs was significantly reduced. Without coating, the scratch made in SMCs had almost disappeared and healed after 24 h. However, when SNAP coating was added, the scratch did not heal completely, with SMC migration ratios of 65% ± 12% (3 μg/mm^2^) and 57% ± 8% (5 μg/mm^2^) (Fig. 3f). In the co-culture experiments of ECs and SMCs (Fig. 3g), the number of endothelial cells increased from 5.61 ± 0.73 × 10^3^ cells/cm^2^ to 5.99 ± 0.75 × 10^3^ cells/cm^2^ after PBM and further increased to 6.54 ± 0.84 × 10^3^ cells/cm^2^ after the introduction of SNAP coating for NO release. In contrast, the number of smooth muscle cells decreased from 7.56 ± 1.32 × 10^3^ cells/cm^2^ to 5.95 ± 0.98 × 10^3^ cells/cm^2^ (PBM) and 5.41 ± 0.81 × 10^3^ cells/cm^2^ (PBM combined with SNAP coating). The ECs/SMCs ratio increased from 0.75 ± 0.07 to 1.23 ± 0.20, which was higher than the 1.02 ± 0.11 when only PBM (Fig. 3h). PBM combined with NO release further promoted the competitive growth of endothelial cells over smooth muscle cells.

**Fig. 4:**
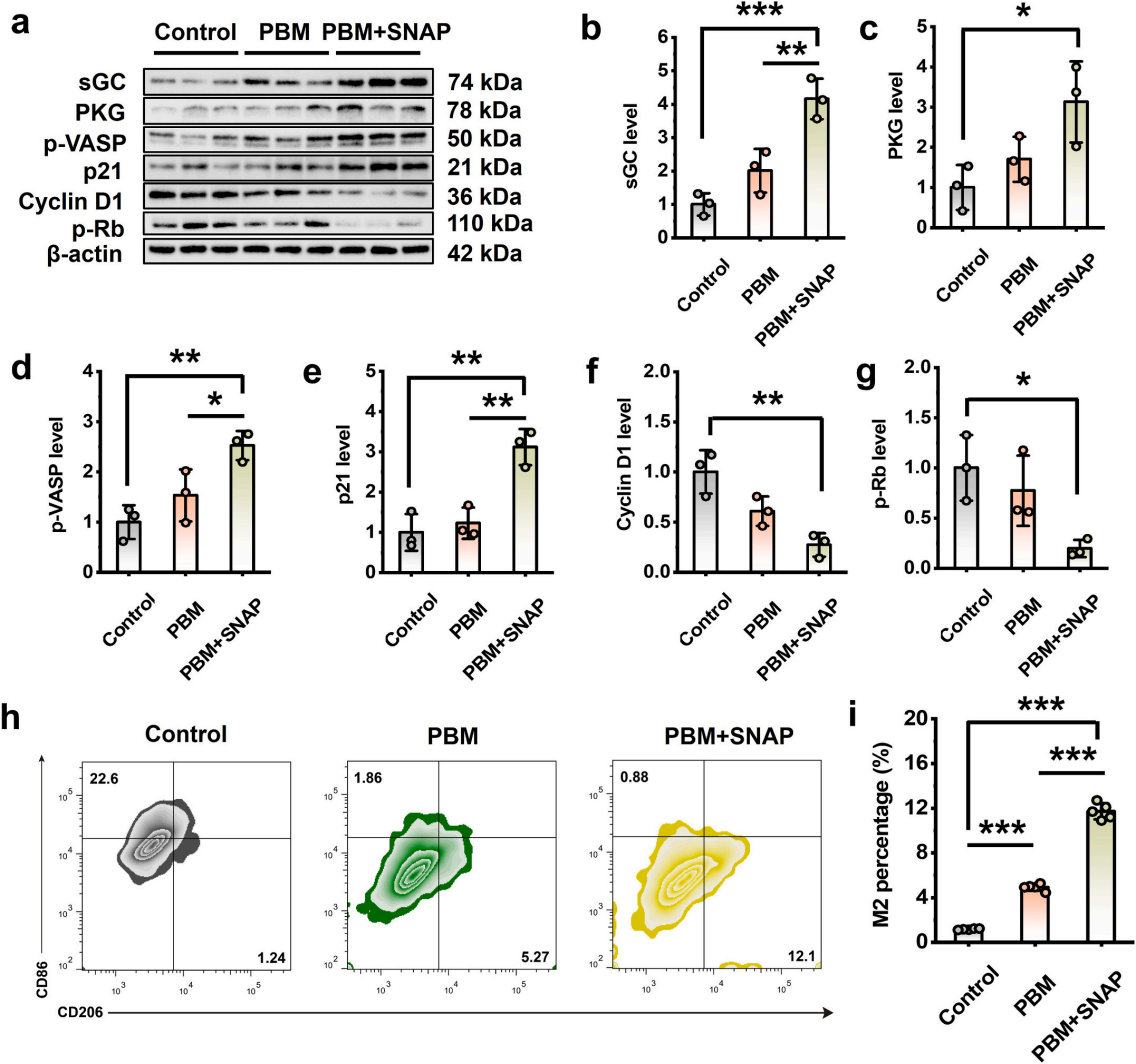
**WB analysis of proteins related to NO/cGMP/PKG pathway and the effect of coating combined with PBM on RAW 264.7 macrophages polarization**. (a) Representative WB bands of sGC、PKG、p-VASP、p21、cyclin D1 and p-Rb in SMCs after PBM and coating treatment. (b-g) WB quantitative data (n = 3). (h) Flow cytometric analysis of RAW 264.7 macrophages after PBM and coating treatment. (i) The proportion of RAW 264.7 macrophages induced into M2-type (n = 5), *p < 0.05, **p < 0.01, ***p < 0.001.

We found that the release of NO activated the NO/cGMP/PKG pathway (Fig. 4a) and activation of PKG exerted an important regulatory effect on SMC activity [52]. After adding SNAP coating, the expression of sGC in SMCs was increased by 316% ± 61% (Fig. 4b) and the expression of PKG was increased by 213% ± 100% (Fig. 4c), with a greater increase compared to only PBM. The NO/cGMP/PKG pathway also activated the vascular dilation-stimulated phosphoprotein p-VASP, inducing SMC relaxation [53]. The p-VASP expression was enhanced by 152% ± 29%, whereas with PBM alone, it was only increased by 53% ± 52% (Fig. 4d). NO regulates the expression of cyclins (such as p21) inhibiting the proliferation of SMCs. After red light triggers NO release, the expression of the cyclin-dependent kinase inhibitor p21 was upregulated by 212% ± 45% (Fig. 4e). The expression of the proliferation-promoting protein cyclin D1 and p-Rb was decreased by 73% ± 12% and 80% ± 9% (Fig. 4f and g) [54,55]. This indicated that NO release effectively blocked the cell cycle through pathway activation, inhibiting their proliferation. This was consistent with the results of the previous SMC proliferation experiment.

To investigate the effect of NO on inflammation, we treated RAW 264.7 macrophages polarized to the M1 phenotype via lipopolysaccharide (LPS) induction with the SNAP coating. According to flow cytometry results, approximately one-quarter of the RAW 264.7 macrophages were successfully induced into pro-inflammatory M1-type cells while the number of anti-inflammatory M2-type cells was extremely scarce. After PBM combined with coating treatment, the number of M1-type cells was significantly reduced, and a considerable number of cells were successfully induced into anti-inflammatory M2-type macrophages (Fig. 4h). After PBM regulation, the percentage of M2-type cells was 4.9% ± 0.3% and this proportion increased to 11.7% ± 0.7% with the addition of SNAP coating (Fig. 4i), demonstrating the role of NO in alleviating inflammatory responses [23,56].

### 3.4 The influence of SNAP coating on inflammation after balloon injury

**Fig. 5:**
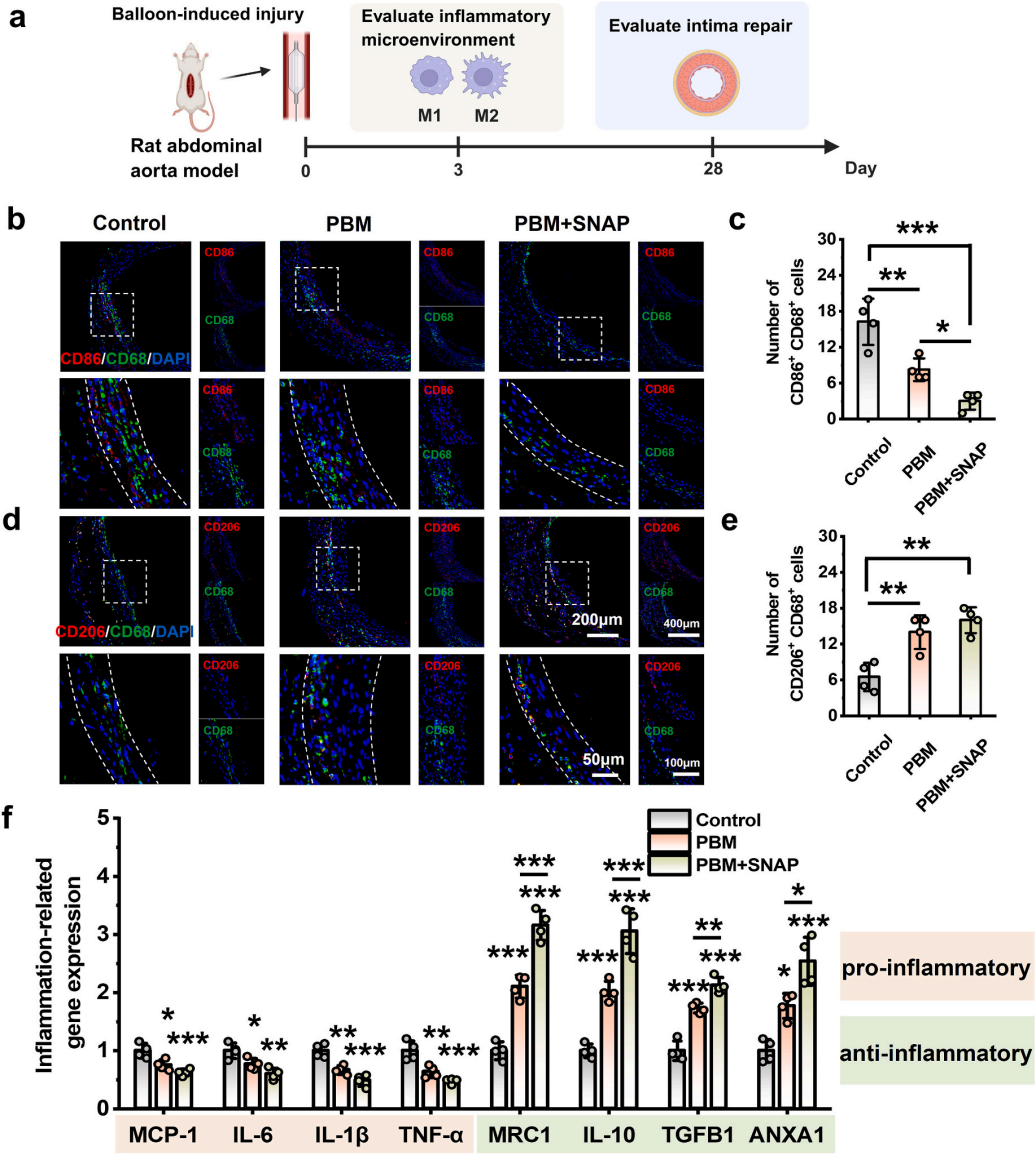
**The effects of coating combined with PBM on inflammation 3 days after balloon injury**. (a) Schematic illustrating balloon injury in the rat abdominal aorta model. (b) CD86 (red) and CD68 (green) double staining. Regions outlined in white are expanded. (c) Corresponding quantification of the numbers of CD86^+^ CD68^+^ cells (n = 4). (d) CD206 (red) and CD68 (green) double staining. (e) Corresponding quantification of the numbers of CD206^+^ CD68^+^ cells (n = 4). (f) RNA expression of inflammation-related genes (n = 4), ∗p < 0.05, ∗∗p < 0.01, ∗∗∗p < 0.001. (For interpretation of the references to color in this figure legend, the reader is referred to the Web version of this article.)

Aiming to evaluate the in vivo repair ability of SNAP coating combined with PBM, rats were subjected to balloon-mediated abdominal aorta injury, which is a well-established mimetic surgical model for balloon angioplasty. In the rat model, a balloon with built-in red light optical fiber was used to give 3.6 J/cm^2^ irradiation(20 mW/cm^2^, 3 min) for PBM therapy and triggering the release of NO from 5 μg/mm^2^ SNAP coating. SNAP coatings on the balloon surface exhibited a NO release capacity similar to sheet coatings (Supplementary Fig. 5). To test the mechanical properties of the coating during use, the coated balloon was subjected to 5 inflation/deflation cycles at 4 atm, 3 min per cycle. Neither macroscopic visual inspection nor SEM imaging revealed any evidence of coating delamination, peeling, cracking, or discoloration after the inflation/deflation cycles (Supplementary Fig. 6). Then we assessed the effect on the inflammatory microenvironment 3 days after balloon injury and evaluated the intima repair effect on day 28 (Fig. 5a).

Controlling inflammation levels after vascular injury in the early stages is crucial for reducing vascular restenosis because inflammation will accelerate the proliferation and migration of SMCs and delay endothelial healing [5,57]. To observe macrophage infiltration in the media layer during the early stages, we performed immunofluorescence staining for total macrophage markers CD68, pro-inflammatory M1 macrophage marker CD86, and anti-inflammatory M2 macrophage marker CD206.3 days after balloon expansion, the number of CD86^+^ CD68^+^ cells was significantly reduced in the PBM combined with SNAP group compared to control group (Fig. 5b and c) while the number of CD206^+^ CD68^+^ cells increased (Fig. 5d and e). This indicated that the combination of PBM and NO release therapy helped to mitigate the inflammatory response. Similarly, qPCR results for inflammation-related genes also supported this conclusion. The combination of PBM and SNAP coating effectively downregulated the expression of pro-inflammatory genes (such as MCP-1, IL-6, IL-1β, TNF-α) and upregulated the expression of anti-inflammatory genes (such as MRC1, IL10, TGFB1, and ANXA-1) after injury. The change was more pronounced compared to PBM alone (Fig. 5f).

**Fig. 6:**
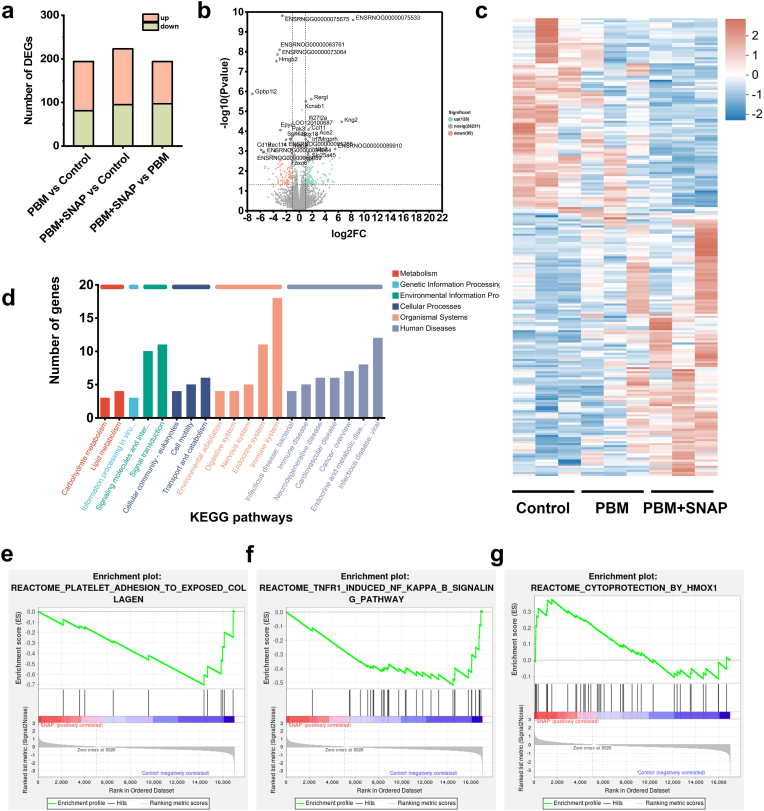
**RNA-seq of abdominal aortas 3 days after balloon injury**. (a) Number of differentially expressed genes (DEGs). (b) Volcano plot of PBM + SNAP vs Control DEGs. (c) Clustering heatmap of differential genes. (d) KEGG annotation analysis of PBM + SNAP vs Control DEGs. (e-g) GSEA analysis of platelet adhesion to exposed collagen, TNF induced NF-κB signaling pathway, cytoprotection by HMOX1 in Control and PBM + SNAP groups.

The RNA-seq of blood vessels 3 days after balloon injury showed a total of 223 DEGs were emerged, including 128 upregulated genes and 95 downregulated genes in PBM combined with SNAP coating versus control group. In PBM versus control group and PBM combined with SNAP coating versus PBM group, the number of DEGs were both 194 (Fig. 6a). We found Kng2 [58], Ace2 [59], Ccl11 [60] and other genes related to vasodilation, inflammatory, angiogenesis were involved in these DEGs (Fig. 6b). The heatmap analysis revealed a significant difference in mRNA expression between different group (Fig. 6c).

Moreover, KEGG annotation analysis further confirmed that differentially expressed genes were significantly enriched in immune system, signal transduction, signaling molecules and interaction, and other aspects after NO release compared to control group (Fig. 6d). Compared to PBM group, KEGG annotation analysis showed DEGs were enriched in signal transduction, infectious disease, cancer overview, and other aspects after NO release (Supplementary Fig. 7). Through GSEA analysis (Fig. 6e–g), we noticed platelet adhesion to exposed collagen and TNF induced NF-κB signaling pathway were downregulated while cytoprotection by HMOX1 signaling pathway was upregulated after NO release. This might be attributed to the anticoagulant and anti-inflammatory effects of NO. In addition, there were differences in aerobic respiration and respiratory electron transport, processing of capped intron-containing pre-mRNA, M phase and other aspects between combined treatment group and control group (Supplementary Fig. 8). As for the group treatment with PBM alone, the differences lay in aspects such as separation of sister chromatids, G2/M checkpoints (Supplementary Fig. 8).

### 3.5 The influence of SNAP coating on intima repair after balloon injury

**Fig. 7:**
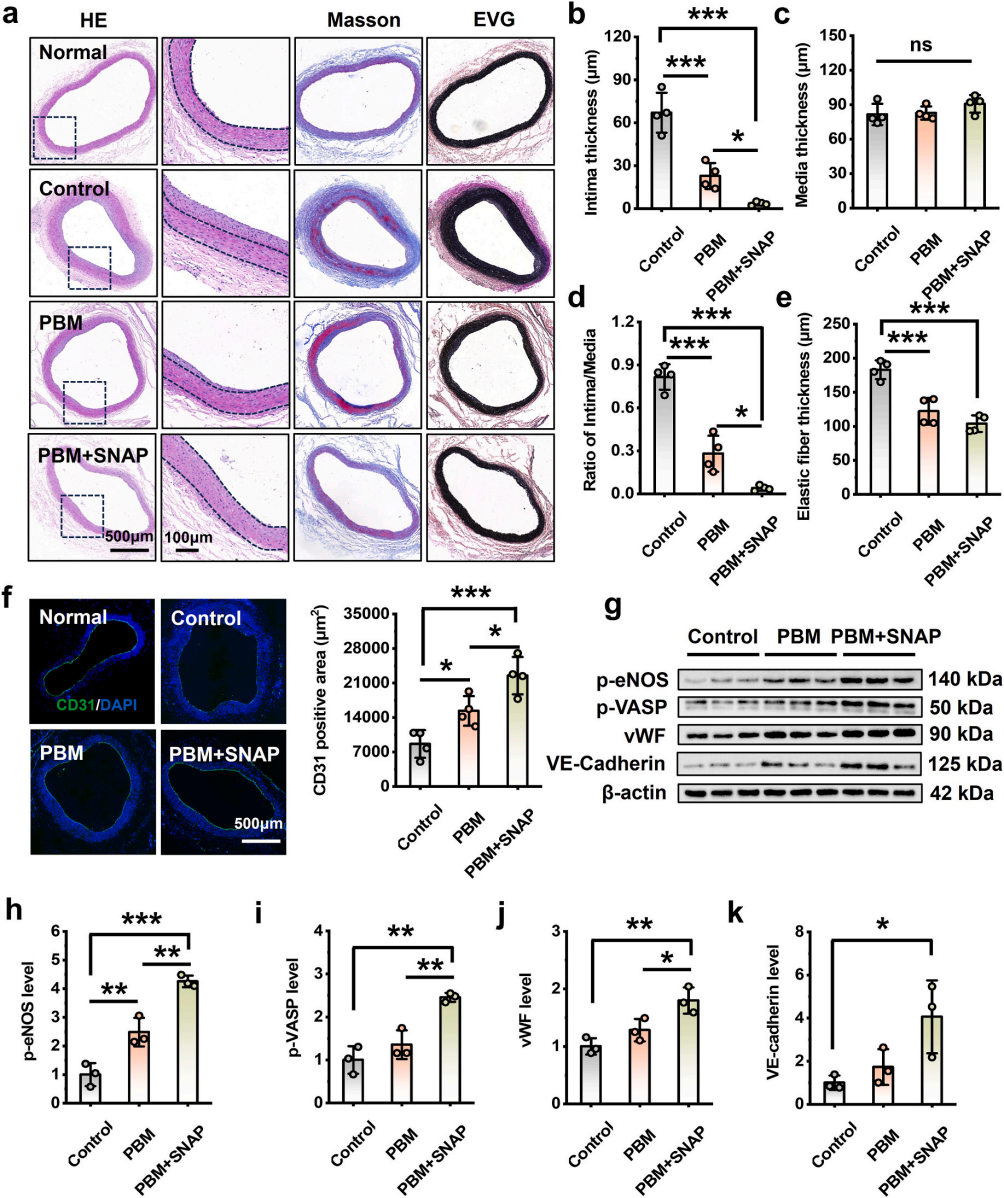
**The effects of coating combined with PBM on intima repair 28 days after balloons injury**. (a) HE, Masson, EVG staining of rat aortas. (b) Quantitative analysis of intima thickness (n = 4). (c) Quantitative analysis of media thickness (n = 4). (d) Quantitative analysis of ratios of intima/media (n = 4). (e) Quantitative analysis of elastic fiber thickness (n = 4). (f) Immunofluorescence staining and quantitative statistical results of CD31 (n = 4), (g) Western blot bands of p-eNOS, p-VASP, vWF, and VE-cadherin proteins, (h-k) Western blot quantitative data (n = 3), ∗p < 0.05, ∗∗p < 0.01, ∗∗∗p < 0.001.

Subsequently, we evaluated the therapeutic effect of PBM combined with SNAP coating on intimal repair 28 days after balloon angioplasty. From the ultrasound examination, there were no significant differences among groups (Supplementary Fig. 9). HE and Masson staining results showed obvious intimal hyperplasia in the abdominal aorta of the control group rats. Mild hyperplasia was present in the PBM group while the experimental group treated with PBM and SNAP coating exhibited almost no hyperplasia, resembling the normal vascular morphology (Fig. 7a). 28 days post-operation, the intimal thickness in the control group rats reached to 67 ± 14 μm. After PBM, the intimal thickness decreased to 23 ± 9 μm, reducing approximately 44 μm. It further decreased to 4 ± 1 μm after PBM combined with SNAP coating, reducing 63 μm (Fig. 7b). The thickness of the vascular media in rats remained relatively unchanged, ranging from approximately 80 to 90 μm (Fig. 7c). The intima/media thickness ratio decreased from 0.82 ± 0.09 to 0.28 ± 0.13 and 0.04 ± 0.02, respectively (Fig. 7d). EVG staining showed the thickness of vascular elastic fiber in control group, PBM group, and PBM combined with coating group were respectively 183 ± 14 μm, 122 ± 19 μm and 104 ± 12 μm (Fig. 7e). Although PBM also effectively inhibited restenosis after balloon angioplasty, the inhibitory effect was further enhanced after the combination with SNAP coating for NO release, demonstrating the potential of SNAP coating in vascular repair.

We also examined the recovery of endothelial cell function post-surgery. CD31 immunofluorescence staining results showed that CD31 expression was increased 1.6-fold after SNAP coating combined with PBM treatment (Fig. 7f), which was greater than that with PBM treatment alone. PBM alone and PBM combined with coating resulted in increases of 148% ± 50% and 326% ± 20% in p-eNOS expression, 35% ± 33% and 146% ± 10% in p-VASP expression, 28% ± 20% and 80% ± 23% in vWF expression, and 73% ± 83% and 306% ± 169% in VE-cadherin expression (Fig. 7g–k). This indicated that NO release effectively enhanced endothelial function recovery after balloon injury and maintained vascular tissue homeostasis.

**Fig. 8:**
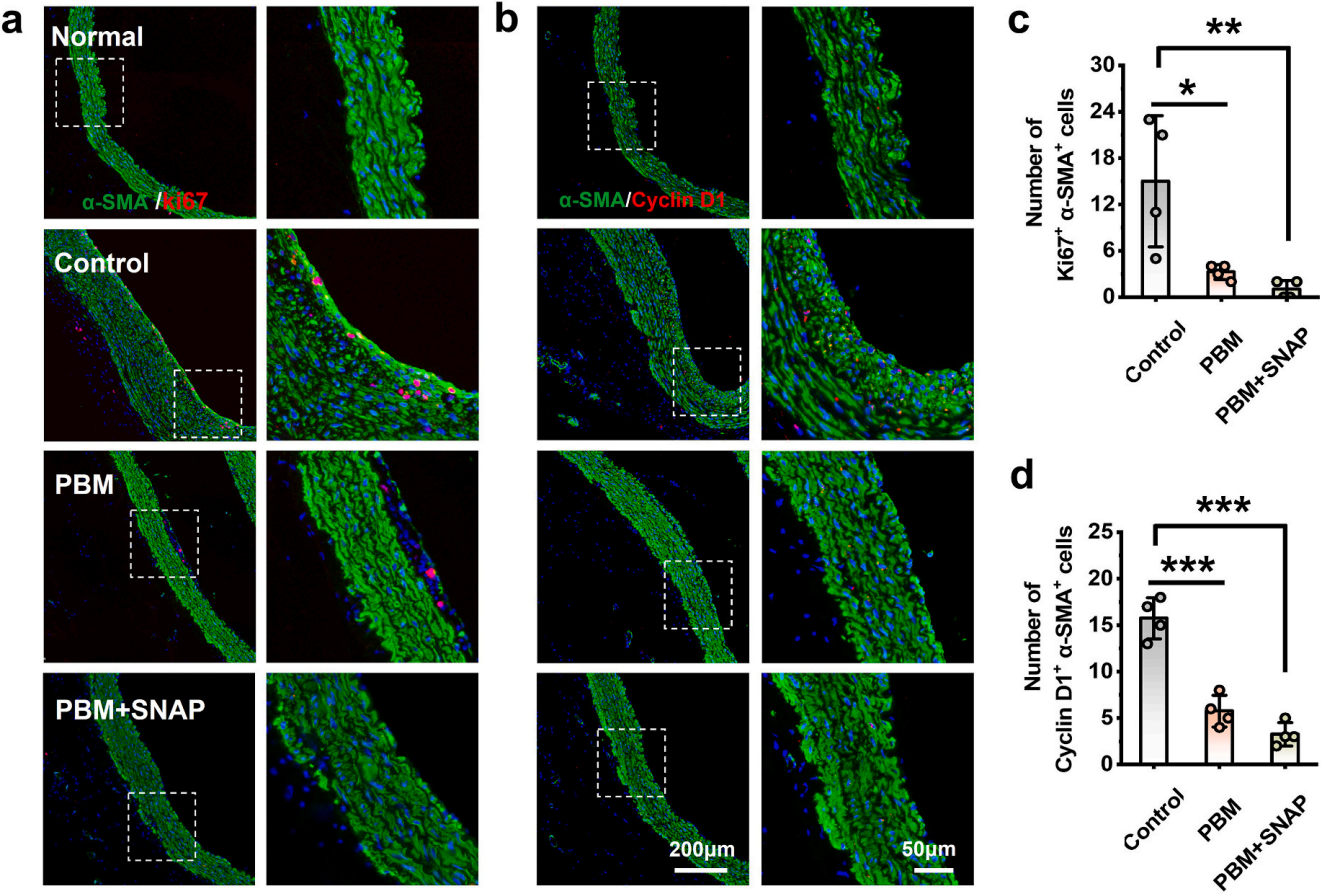
**The effects of coating combined with PBM on SMC proliferation 28 days after balloon injury**. (a) Representative Ki67 (red) and α-SMA (green) double staining of aortas from different groups. Regions outlined in white are expanded. (b) Representative Cyclin D1 (red) and α-SMA (green) double staining of aortas from different groups. Regions outlined in white are expanded. (c) Corresponding quantification of the numbers of Ki67^+^ α-SMA^+^ cells (n = 4). (d) Corresponding quantification of the numbers of Cyclin D1^+^ α-SMA^+^ cells (n = 4), ∗p < 0.05, ∗∗p < 0.01, ∗∗∗p < 0.001. (For interpretation of the references to color in this figure legend, the reader is referred to the Web version of this article.)

To detect the proliferation status of smooth muscle cells 28 days post-surgery, we performed staining for two classical proliferation markers, Ki67 and Cyclin D1. In the control group, the intima region had obvious co-expression of proliferation markers Ki67 and Cyclin D1 with the SMC marker α-SMA (Fig. 8a–d). In contrast, the PBM group exhibited only a small number of Ki67^+^ α-SMA^+^ and Cyclin D1^+^ α-SMA^+^ cells. After PBM combined with coating treatment, the number of proliferating-positive smooth muscle cells was further reduced to almost none. This was consistent with previous finding that rats treated with SNAP coating and PBM had minimal intimal hyperplasia.

## 4 Discussion

Many NO-delivery strategies have also been developed and investigated for biomedical applications. In wound healing, NO release materials are applied in angiogenesis, cell proliferation and collagen deposition [61]. In anti-restenosis therapy, NO release surfaces are used to thwart thrombus, inhibit the SMCs growth and accelerate endothelialization [62,63]. Several coating methods for NO release have been reported, such as catalytic SeCA systems [64], SNAP-incorporated polymer blends [65], and electrospinning [66]. These studies focus on sustained NO release from vascular stents and grafts via passive or catalytic mechanisms [67]. Most of these systems rely on UV/blue light (which carries cytotoxicity risks) or catalytic activity that depends on endogenous substrate availability, and none have integrated photobiomodulation or been specifically designed for balloon catheters used in acute post-angioplasty injury. UV and blue light face two major limitations in biomedical applications: limited tissue penetration depth and potential cytotoxicity. In recent years, red light or NIR-triggered NO donor materials have emerged increasingly; nevertheless, most of them still rely on up conversion nanoparticles (UCNPs) to convert NIR light into UV/blue light for indirect activation, which raises concerns regarding material toxicity and complex preparation procedures [68]. The entire process of balloon dilation is very short. How to achieve the on-demand release of NO within a short time is crucial, and response technology is a good solution to this problem. Our study presents a red-light-responsive SNAP/shellac coating engineered explicitly for balloon catheters. We synergistically combine PBM (which enhances mitochondrial respiration) with exogenous photo-triggered NO release, achieving spatiotemporal control, avoiding UV/blue-light toxicity, and addressing the unmet need for acute vascular repair after balloon angioplasty.

We prepared a drug coating of the photosensitive NO donor SNAP by ultrasonic spraying. This coating enabled controlled NO release under red light irradiation and exhibited good anticoagulant properties. The biological function of NO in organisms depends on the concentration of NO. High concentrations of NO in the tissue will enhance the cell toxicity, inhibit cell proliferation and accelerate the cell apoptosis. The basal NO flux from healthy vascular endothelium is approximately 0.05–0.4 nmol/cm^2^/min [69,70]. Our coating releases NO at rates of 4.8 nmol/cm^2^/min upon red light irradiation (5 μg/mm^2^). While this exceeds the physiological level, it is intentional for therapeutic purposes: to provide an acute, on-demand NO burst that rapidly inhibits platelet adhesion and kick-starts endothelial repair following balloon injury. Importantly, this NO level is transient, light-dependent, and below cytotoxic thresholds, as supported by our cell viability and in vivo data. After releasing NO, the SNAP coating effectively promoted EC migration and proliferation, increasing the proliferation rate and the number of tube formations, which was of positive significance for the recovery of endothelial cell function. Meanwhile, the coating inhibited the proliferation and migration activity of SMCs by activating NO/cGMP/PKG pathway. In vivo experiments, we used rat abdominal aorta balloon injury model to mimic mechanical overstretch and endothelial denudation. Though it was not exactly the same as pathological process of dilating a pre-existing atherosclerotic stenosis as occurs in clinical balloon angioplasty, it represented neointimal formation following injury. Mechanistic overlap between PBM-triggered endogenous NO release and exogenous NO from SNAP photolysis was considered. PBM alone is known to generate NO through multiple pathways [71,72]. Endogenous NO release from PBM alone was modest based on our previous report while SNAP coating provided a high-flux exogenous NO source upon red light irradiation, which was substantially greater than what PBM alone could generate from endogenous stores. We therefore propose that the two mechanisms are predominantly additive, with PBM potentially providing a preconditioning effect while SNAP delivers the therapeutic NO required for rapid platelet inhibition and endothelial repair. Importantly, our experimental design comparing PBM alone versus PBM + SNAP groups demonstrates that the combined approach yields superior outcomes.

Of course, our study also has several limitations. SNAP has a certain degree of hydrophilicity. Although we added shellac to the coating, part of the SNAP still dissolves. There is still room for improvement in the stability of the coating and the selection of excipients. While this study demonstrates the certain efficacy of SNAP coating in attenuating the development of intimal hyperplasia at intermediate time points, it is acknowledged that long-term outcome data are absent. Restenosis is a chronic process, and the long-term durability of the therapeutic effect warrants further investigation. Future studies employing extended observation periods (90 or 180 days) in larger animal models are essential to confirm the sustained suppression of neointimal growth and the long-term safety profile of this approach. The failure to implement double-blindness is also an aspect that can be improved. More additional control groups (SNAP only without light, shellac only) would be included to complement the current findings in the future. Further biocompatibility testing including evaluation of cytotoxicity, sensitization, systemic toxicity, genotoxicity, and local effects should be performed as required by the FDA if promoted for clinical application.

Despite research demonstrating the benefits of NO therapy in rat models, successful translation to more clinically relevant large animal models and ultimately to human vascular pathologies still has a long way to go. In this context, the red light-triggered NO-releasing coating described here offers a potential strategy to address translational barriers. Unlike conventional NO donors that rely on passive diffusion or enzymatic activation, our approach enables spatiotemporally controlled NO delivery via external red light irradiation. This feature is particularly relevant for translation, as red light can be delivered intravascularly using endovascular devices, allowing on-demand NO release at the target lesion. Importantly, because light-triggered NO release is independent of endogenous enzymes or pH gradients, it may offer more consistent release kinetics, potentially enhancing the possibility of clinical translation. We hope this research can facilitate the innovation and development of balloon devices, providing a possible method for endothelialization.

## 5 Conclusion

In this study, we developed a photobiomodulation and photo-triggered drug release strategy to promote vascular intimal healing following balloon injury. A drug coating of the photosensitive NO donor SNAP was fabricated via ultrasonic spraying technology. The resulting coating enabled controlled, light-triggered NO release and exhibited excellent anticoagulant properties. After releasing NO, the SNAP coating effectively promoted EC migration and proliferation, increasing proliferation by approximately 20% and enhancing tube formation by 3-fold, which was of positive significance for the recovery of endothelial cell function. Meanwhile, the coating inhibited the proliferation and migration activity of SMCs, with a 43% decrease in migration ratio. In vivo, red light triggered NO release further alleviated vascular inflammation and restenosis. Notably, the combined therapeutic approach integrating PBM with the SNAP coating demonstrated superior efficacy compared to PBM alone, highlighting the potential for vascular repair.

## CRediT authorship contribution statement

**Shu-jie Jin:** Investigation, Methodology, Validation, Visualization, Writing – original draft. **Chen-ze Deng:** Data curation. **Rou-ye Wang:** Data curation, Visualization. **Fan Jia:** Investigation. **Pei-hong Ji:** Conceptualization. **Ya Peng:** Data curation, Validation. **Ke-feng Ren:** Conceptualization, Funding acquisition, Project administration, Resources, Supervision, Writing – review & editing. **Jian-min Liu:** Methodology, Resources, Supervision. **You-xiang Wang:** Conceptualization, Funding acquisition, Writing – review & editing. **Jian Ji:** Funding acquisition.

## Declaration of competing interest

The authors declare that they have no known competing financial interests or personal relationships that could have appeared to influence the work reported in this paper.

## Data Availability

Data will be made available on request.
