# Supplementary material for: Photo-triggered NO combined with photobiomodulation promotes endothelial repair after balloon angioplasty
Source: Mater Today Bio. 2026 Jul 29;40:103497. doi: 10.1016/j.mtbio.2026.103497 (PMC13470320; doi:10.1016/j.mtbio.2026.103497)
Supplement: Supplementary file 1 [file mmc1.docx]

**Supplementary information**

**Photo-Triggered NO Combined with Photobiomodulation Promotes Endothelial Repair after** **Balloon Angioplasty**

Shu-jie Jin,^1^ Chen-ze Deng,^1^ Rou-ye Wang,^1^ Fan Jia,^2^ Pei-hong Ji,^3^ Ya Peng,^4^ Ke-feng Ren,^1,2^* Jian-min Liu,^5^* You-xiang Wang^1^* and Jian Ji^1,6^

1. MOE Key Laboratory of Macromolecular Synthesis and Functionalization, Department of Polymer Science and Engineering, Zhejiang University, Hangzhou 310058, China
2. Key Laboratory of Cardiovascular Intervention and Regenerative Medicine of Zhejiang Province, Department of Cardiology, Sir Run Run Shaw Hospital, Zhejiang University, Hangzhou 310016, China
3. Hangzhou Matrix Medical Technology Co. Ltd, Hangzhou 311100, China
4. Neurosurgery Department of the Third Affiliated Hospital of Soochow University, Changzhou 213003, China
5. Neurovascular Center, Changhai Hospital, Naval Medical University, Shanghai 200433, China
6. State Key Laboratory of Transvascular Implantation Devices, the Second Affiliated Hospital Zhejiang University School of Medicine, Hangzhou 310009, China

*Correspondence: renkf@zju.edu.cn, liu118@vip.163.com, yx_wang@zju.edu.cn

**This file contains the following Supplementary Figures:**

Supplementary Fig.1-Thickness of SNAP coatings.

Supplementary Fig.2- The leaching of 5 μg/mm^2^ SNAP/shellac coating in PBS.

Supplementary Fig.3- The water contact angle of the coating.

Supplementary Fig.4-Effect of SNAP coatings on cell activity without red light irradiation.

Supplementary Fig.5-SNAP coating and NO release on balloon surface.

Supplementary Fig.6- Inflation/deflation test of balloon coating.

Supplementary Fig.7-Volcano plot and KEGG annotations analysis of DEGs.

Supplementary Fig.8- GSEA analysis of genes.

Supplementary Fig.9-Color Doppler ultrasound of rat aortas 28 days after balloon injury.

**Supplementary Fig.1 Thickness of SNAP coatings.**

**Supplementary Fig. 2** **The leaching of 5 μg/mm^2^ SNAP/shellac coating in PBS.** (a) Ultraviolet absorption spectra obtained at different leaching times. (b) The percentage of total SNAP loaded that diffused into the buffer (n = 3).

**Supplementary Fig. 3 The water contact angle of the coating.**

**Supplementary Fig. 4** **Effect of SNAP coatings on cell activity without red light irradiation.** (a) Effect of SNAP coatings on EC activity without red light irradiation (n = 9). (b) Effect of SNAP coatings on SMC activity without red light irradiation (n = 9).

**Supplementary Fig. 5 SNAP coating and NO release on balloon surface.** (a) Image of 5 μg/mm^2^ SNAP/Shellac coating on balloon surface. (b) NO release capacity of different drug-loading densities coatings on balloon surface under red light irradiation (n = 3).

**Supplementary Fig.6 Inflation/deflation test of balloon coating.**

**Supplementary Fig. 7 Volcano plot and** **KEGG annotations analysis of DEGs.** The pictures are arranged from top to bottom as follows: PBM vs Control, PBM+SNAP vs Control, PBM+SNAP vs PBM.

**Supplementary Fig. 8** **GSEA analysis of genes.**

**Supplementary Fig. 9** **Color Doppler ultrasound of rat aortas 28 days after balloons injury.** The images above showed blood flow velocity while the images below showed vessel diameter.
